# Association of anthropometric measures of obesity and physical activity with cardio-vascular diseases among older adults: Evidence from a cross-sectional survey, 2017–18

**DOI:** 10.1371/journal.pone.0260148

**Published:** 2021-12-15

**Authors:** Snigdha Banerjee, Pradeep Kumar, Shobhit Srivastava, Adrita Banerjee

**Affiliations:** 1 International Institute for Population Sciences, Mumbai, India; 2 Population Council, India Country Office, India Habitat Centre, New Delhi, India; 3 Research and Innovation, MAMTA Health Institute for Mother and Child, New Delhi, India; Nazarbayev University School of Medicine, KAZAKHSTAN

## Abstract

**Background:**

With the increase in elderly population, the risk of cardiovascular diseases (CVD) among Indian older adults is also increasing. The present paper tries to assess how different anthropometric measures of obesity and physical activity affects cardiovascular disease risk among older adults in India.

**Methods:**

The data from Longitudinal Ageing Study in India (LASI) has been used. The total sample size for the present study is 31,464 older adults aged 60 years and above. Chi-square test and binary logistic regression has been used to measure the association of obesity measures and CVD prevalence.

**Results:**

About 35.2% (n = 11,058) of the older adults suffered from CVD. Moreover, 22.2% (n = 6,217) of the older adults were obese/overweight, 23.7% (n = 6,651) had high risk waist circumference and 77.0% (n = 21,593) had high risk waist-Hip ratio. The likelihood of CVD was 60%, 50%, and 34% significantly higher among older adults who were obese/overweight [Adjusted odds ratio (AOR): 1.60; CI: 1.48–1.72], had high risk waist circumference [AOR: 1.50; CI: 1.39–1.62] and high risk waist-Hip ratio [AOR: 1.34; CI: 1.25–1.44], respectively compared to older adults with normal BMI and those who do not have a high risk waist circumference and high risk waist hip ratio. Moreover, older adults who never did physical activity had 22 per cent higher risk of CVD than those who did frequent [AOR: 1.22; CI: 1.13–1.32] physical activity.

**Conclusion:**

The burden of overweight and obesity along with physical inactivity increases the risk of CVD in older adults. These findings highlight the urgent need for framing direct and indirect strategies to control obesity in order to reduce the burden of CVD among older adults in India.

## Introduction

India is experiencing an increase in the elderly population as it is advancing in stages of demographic transition. According to the Indian Census, 2011, the number of people aged 60 years and above had reached nearly 104 million, accounting for 8.6% of the nation’s total population [1]. According to United Nations projection, the 60+ population will increase to 11% by 2025 and 19% by 2050; However, demographic transition in India has been accompanied by the epidemiological transition and the disease profile has witnessed a phenomenal change from communicable disease to non-communicable diseases [2]. This demographic transition has brought many people to the age group where cardiovascular diseases (CVDs) manifest. Older adults have a high risk of the dual burden of both communicable and non-communicable diseases [3].

CVDs comprises coronary heart disease (CHD), strokes, rheumatic heart disease (RHD), cardiomyopathy, and other heart diseases, which represent the leading cause of death globally [4, 5]. Over three-quarters of CVD deaths take place in low- and middle-income countries. In 2017, the Global Burden of Disease Study revealed that China, India, and South Africa contribute an increasing proportion of global CVDs deaths [6]. Compared with the people of the European country, CVD affects Indians at least a decade earlier and in their most productive midlife years. For example, in Western populations, only 23 per cent of CVD deaths occur before the age of 70; in India, this number is 52 per cent [7]. Therefore, case fatality attributable to CVD in low-income countries, including India, appears much higher than in the middle- and high-income countries [8]. CVD was taking an estimated 17.9 million lives each year [7]. According to a WHO report, the current age-standardized CVD mortality rates among males and females in India are 400 and 281 per 100,000, respectively [9]. In India, the age-standardized annual stroke incidence rate is 154 per 100 000 per year. However, the stroke incidence and stroke-related case fatality rates in India are higher relative to Western industrialized nations. The rates are significantly higher among women [10]. The World Health Organization has estimated that India experienced more than half of the non-communicable disease burden. It was estimated that with the current CVD burden, India would lose more than two hundred billion dollar from the loss of productivity and spending on health care [11, 12].

However, the most important behavioural risk factors of heart disease and stroke are unhealthy diet, physical inactivity, harmful use of alcohol, tobacco, obesity, abdominal obesity (high waist-hip ratio), low socio-economic position, and having a family record of premature cardiovascular disease [13]. It has been estimated that inadequate physical activity is responsible for more than 30 per cent of deaths due to coronary heart disease and diabetes [14]. On the other hand, obesity is also a growing health problem in developed and developing countries, strongly related to major cardiovascular risk factors [15, 16]. World Health Organization suggests that all individuals should be strongly encouraged to reduce total fat and saturated fat intake. Physical activities are also very much necessary to reduce the risk of CVD. An individual should lose weight through a combination of a reduced energy diet and increased physical activities. Therefore, cessation of tobacco use [17], reduction of salt in the diet, consuming a healthy diet [18], regular physical activity and avoiding tobacco have been shown to reduce the risk of cardiovascular disease [7].

Despite acknowledging CVD as a significant public health problem, India’s access to cardiovascular care remains relatively low as reflected in poor detection, treatment, and adherence to evidence-based treatment options among Indians [19]. India’s primary health system has mostly been geared toward the management of communicable diseases. Hence, special attention is required for the innovative integration of CVD preventive care in the primary healthcare system of India [11]. In India, older adults are at more significant health risks owing to their peculiar socio-economic and cultural characteristics [20], including low nutritional intake [21], poor quality of health facilities and health care access resulting in one of the highest out of pocket health care expenditure [2]. The risk factor of cardiovascular diseases among Indian older adults is, however, relatively under-explored. In this context, any insightful assessment of the CVD and association of various covariates among older adults become pertinent [1]. With the increase in overweight and obesity the risk of CVD also increases. Though obesity is a pandemic phenomenon in the developed parts but has also reached heights in developing country and India is no exception to this [22]. There is a need to explore the association between obesity measures, socio-economic determinants and the risk of CVD among older adults. This information is also important from policy and program perspectives to bring future actions on elderly issues [23]. Thus, our study attempts to examine the risk factors of CVD among older adults in India.

## Method and materials

### Data

Data for this study was utilized from the recent release of Longitudinal Ageing Study in India (LASI) wave 1. LASI is a full-scale national survey of scientific investigation of the health, economic, and social determinants and consequences of population ageing in India, conducted in 2017–18. The LASI is a nationally representative survey of over 72000 older adults aged 45 and above across all states and union territories of India. The main objective of the survey is to study the health status and the social and economic well-being of older adults in India. LASI adopted a multistage stratified area probability cluster sampling design to arrive at the eventual units of observation: older adults age 45 and above and their spouses irrespective of age. The survey adopted a three-stage sampling design in rural areas and a four-stage sampling design in urban areas. In each state/union territory (UT), the first stage involved the selection of Primary Sampling Units (PSUs), that is, sub-districts (Tehsils/Talukas), and the second stage involved the selection of villages in rural areas and wards in urban areas in the selected PSUs. In rural areas, households were selected from selected villages in the third stage. However, sampling in urban areas involved an additional stage. Specifically, in the third stage, one Census Enumeration Block (CEB) was randomly selected in each urban area. In the fourth stage, households were selected from this CEB. The detailed methodology, with the complete information on the survey design and data collection, was published in the survey report [24]. The present study is conducted on the eligible respondent’s aged 60 years and above. The total sample size for the present study is 31,464 older adults aged 60 years and above.

### Variable description

#### Outcome variable

The outcome variable “cardiovascular diseases (CVD)” was binary in nature. The variable was measured using the question “Has any health professional ever diagnosed you with hypertension or heart disease or stroke?” The response was categorized as 0 “no” and 1 “yes”. Such patient-reported outcome measures (PROM) do have their salience in this particular context [25].

#### Explanatory variable

Overweight/obesity was categorized as no and yes. The respondents having body mass index of 25 and above were categorized as obese/overweight [26]. High risk waist circumference was categorized as no and yes. Male and female who have waist circumferences of more than 102 cm and 88 cm respectively were considered having high risk waist circumference [27]. High risk waist-hip ratio (WHR) was categorized as no and yes. Male and female who have waist-hip ratio of more than equal to 0.90 and 0.85 cm respectively were considered having high risk waist-hip ratio [27]. Physical activity status was categorized as frequent (every day), rare (more than once a week, once a week, one to three times in a month) and never. The question through which physical activity was assessed was “How often do you take part in sports or vigorous activities, such as running or jogging, swimming, going to a health centre or gym, cycling, or digging with a spade or shovel, heavy lifting, chopping, farm work, fast bicycling, cycling with loads”? [24].

Age was categorized as young old (60–69 years), old-old (70–79 years) and oldest-old (80+ years) [28]. Sex was categorized as male and female. Education was categorized as no education/primary schooling not completed, primary completed, and secondary completed and higher & above. Marital status was categorized as currently married, widowed and others (separated/never married/divorced). Working status was categorized as working, retired and not working. Tobacco and alcohol consumption was categorized as no and yes.

Monthly per capita expenditure (MPCE) quintile was estimated using household consumption data. A set of 11 and 29 questions on the expenditures incurred on non-food and food items, respectively, was used to canvas the sample households. Food expenditure was collected based on a reference period of seven days, and non-food expenditure was collected based on reference periods of 30 days and 365 days. Food and non-food expenditures have been standardised to the 30-day reference period. The monthly per capita consumption expenditure (MPCE) is computed and used as the summary measure of consumption [24]. The variable was then divided into five quintiles i.e., from poorest to richest. Religion was categorized as Hindu, Muslim, Christian and Others. Caste was categorized as Scheduled Tribe, Scheduled Caste, Other Backward Class and others. The Scheduled Caste include a group of population which is socially and financially/economically segregated by their low status as per Hindu caste hierarchy [29]. The Scheduled Castes (SCs) and Scheduled Tribes (STs) are among the most disadvantaged socio-economic groups in India. The OBC’s are the group of people who were identified as “educationally, economically and socially backward”. The OBC’s are considered low in the traditional caste hierarchy but are not considered as untouchables [30]. The “other” caste category are identified of having higher social status. Place of residence was categorized as rural and urban. Region was categorized as North, Central, East, Northeast, West and South.

*Statistical analysis*. Descriptive statistics along with bivariate analysis was used in the present paper. Chi-square test [31] was used to measure the significance level for CVD prevalence among older adults by their background characteristics. Additionally, binary logistic regression analysis [32] was used to establish the association between outcome variable (CVD) and other explanatory variables.

The binary logistic regression model is usually put into a more compact form as follows:

$$Logit\;\left[P\left(Y=1\right)\right]=\beta_{0}+\beta *X$$

The parameter *β*_0_ estimates the log odds of the CVD for the reference group, while *β* estimates the maximum likelihood, the differential log odds of the CVD associated with set of predictors X, as compared to the reference group. The variance inflation factor (VIF) was used to measure collinearity and it was observed that there was no collinearity found in the variable used [33].

The multivariate analysis had four models to explain the adjusted estimates. Model-1 provide the adjusted estimates for the explanatory variables. Model-2, model-3 and model-4 provide the interaction effects [34–36] for obesity indicators and physical activity with CVD among older adults. The analysis was done using Stata 14 [37].

## Results

### Socio-demographic and economic profile of study population (Table 1)

**Table 1:** Socio-demographic and economic profile of older adults in India.

Background characteristics	Sample	%
**Obese/overweight** ^a^		
No	21833	77.8
Yes	6217	22.2
**High risk waist circumference** ^a^		
No	21399	76.3
Yes	6651	23.7
**High risk waist-Hip ratio** ^a^		
No	6434	23.0
Yes	21593	77.0
**Physical activity status**		
Frequent	5651	18.0
Rare	4023	12.8
Never	21790	69.3
**Age (in years)**		
60–69	18410	58.5
70–79	9501	30.2
80+	3553	11.3
**Sex**		
Male	14931	47.5
Female	16533	52.6
**Education**		
No education/primary not completed	21380	68.0
Primary completed	3520	11.2
Secondary completed	4371	13.9
Higher and above	2191	7.0
**Marital status**		
Currently married	19391	61.6
Widowed	11389	36.2
Others	684	2.2
**Working status**		
Working	9680	30.8
Retired	13470	42.8
Not working	8314	26.4
**Tobacco consumption**		
No	18964	60.3
Yes	12500	39.7
**Alcohol consumption**		
No	26924	85.6
Yes	4540	14.4
**MPCE**		
Poorest	6829	21.7
Poorer	6831	21.7
Middle	6590	21.0
Richer	6038	19.2
Richest	5175	16.5
**Religion**		
Hindu	25871	82.2
Muslim	3548	11.3
Christian	900	2.9
Others	1145	3.6
**Caste**		
Scheduled Caste	5949	18.9
Scheduled Tribe	2556	8.1
Other Backward Class	14231	45.2
Others	8729	27.7
**Place of residence**		
Rural	22196	70.6
Urban	9268	29.5
**Region**		
North	3960	12.6
Central	6593	21.0
East	7439	23.6
Northeast	935	3.0
West	5401	17.2
South	7136	22.7
**Total**	31,464	100.0

^a^ The sample may differ as all older adults did not gave consent for the measurements.

About 22 per cent of older adults were obese/overweight, one-fourth of older adults had high risk waist circumference, and 77 per cent of older adults had high risk waist-hip ratio. Only 18 per cent of older adults did frequent physical activity. About 59 per cent of older adults belonged to 60–69 years age group, only seven per cent had higher education, and 30 per cent of older adults were working. Two-fifth of older adults used tobacco and nearly 14 per cent of older adults consumed alcohol.

### Prevalence of CVD among older adults in India (Table 2)

**Table 2:** Percentage of older adults suffering from CVD by their background characteristics in India.

Background characteristics	%	p-value
**Obese/overweight**		<0.001
No	28.9	
Yes	56.7	
**High risk waist circumference**		<0.001
No	28.4	
Yes	56.3	
**High risk waist-Hip ratio**		<0.001
No	25.3	
Yes	37.9	
**Physical activity status**		<0.001
Frequent	27.7	
Rare	26.0	
Never	38.8	
**Age (in years)**		<0.001
60–69	33.3	
70–79	37.9	
80+	37.3	
**Sex**		<0.001
Male	31.1	
Female	38.8	
**Education**		<0.001
No education/primary not completed	30.7	
Primary completed	41.3	
Secondary completed	44.6	
Higher and above	50.0	
**Marital status**		<0.001
Currently married	33.2	
Widowed	38.9	
Others	27.6	
**Working status**		<0.001
Working	22.9	
Retired	37.9	
Not working	45.0	
**Tobacco consumption**		<0.001
No	39.3	
Yes	28.9	
**Alcohol consumption**		<0.001
No	36.3	
Yes	28.1	
**MPCE**		<0.001
Poorest	28.0	
Poorer	30.9	
Middle	34.3	
Richer	39.9	
Richest	45.8	
**Religion**		<0.001
Hindu	34.1	
Muslim	38.6	
Christian	39.4	
Others	45.1	
**Caste**		<0.001
Scheduled Caste	30.7	
Scheduled Tribe	19.3	
Other Backward Class	35.1	
Others	42.8	
**Place of residence**		<0.001
Rural	29.4	
Urban	48.9	
**Region**		<0.001
North	40.7	
Central	22.9	
East	33.9	
Northeast	38.5	
West	38.9	
South	41.3	
**Total**	35.2	

Overall, nearly 35 per cent of older adults suffered from CVD in India. The prevalence of CVD was significantly higher among older adults who were obese/overweight (56.7 per cent), had high risk waist circumference (56.3 per cent), and those who had high risk waist-Hip ratio (37.9 per cent). Moreover, it was more prevalent among older adults who never did physical activity (38.8 per cent). Female older adults reported significantly higher prevalence of CVD than male counterparts (38.8 per cent vs. 31.1 per cent). Women’s education and MPCE has positive association with the prevalence of CVD among older adults. For instance, as increase the level of education and MPCE, the prevalence of CVD also increased. The prevalence of CVD was significantly higher among older adults who were retired (37.9 per cent)/not working (45 per cent) compared to those who were working. Older adults who lived in urban areas suffered more from CVD than rural counterparts (48.9 per cent vs. 29.4 per cent). CVD was more prevalent in North (40.7 per cent) and South region (41.3 per cent) and it was lowest in Central region of India (22.9 per cent).

### Estimates from logistic regression analysis for CVD among older adults in India (Table 3)

**Table 3:** Logistic regression estimates for CVD among older adults by their background characteristics in India.

Background characteristics	Model-1	Model-2	Model-3	Model-4
	AOR 95% CI	AOR 95% CI	AOR 95% CI	AOR 95% CI
**Obese/overweight**				
No	Ref.		Ref.	Ref.
Yes	1.60*(1.48,1.72)		1.64*(1.51,1.80)	1.59*(1.48,1.72)
**High risk waist circumference**				
No	Ref.	Ref.		Ref.
Yes	1.50*(1.39,1.62)	1.52*(1.39,1.65)		1.50*(1.39,1.62)
**High risk waist-Hip ratio**				
No	Ref.	Ref.	Ref.	
Yes	1.34*(1.25,1.44)	1.34*(1.25,1.44)	1.33*(1.21,1.41)	
**Physical activity status**				
Frequent	Ref.			
Rare	1.00(0.91,1.11)			
Never	1.22*(1.13,1.32)			
**Age (in years)**				
60–69	Ref.	Ref.	Ref.	Ref.
70–79	1.22*(1.15,1.30)	1.22*(1.15,1.30)	1.22*(1.15,1.30)	1.22*(1.15,1.30)
80+	1.19*(1.08,1.30)	1.19*(1.08,1.30)	1.19*(1.08,1.30)	1.19*(1.08,1.31)
**Sex**				
Male	Ref.	Ref.	Ref.	Ref.
Female	1.02(0.94,1.10)	1.02(0.94,1.10)	1.02(0.94,1.10)	1.02(0.94,1.10)
**Education**				
No education/primary not completed	Ref.	Ref.	Ref.	Ref.
Primary completed	1.26*(1.16,1.36)	1.26*(1.16,1.36)	1.26*(1.16,1.36)	1.26*(1.16,1.37)
Secondary completed	1.24*(1.15,1.35)	1.24*(1.15,1.35)	1.24*(1.15,1.35)	1.24*(1.15,1.35)
Higher and above	1.36*(1.22,1.52)	1.36*(1.22,1.52)	1.36*(1.22,1.52)	1.36*(1.22,1.52)
**Marital status**				
Currently married	Ref.	Ref.	Ref.	Ref.
Widowed	1.10*(1.03,1.17)	1.10*(1.03,1.17)	1.11*(1.04,1.19)	1.10*(1.03,1.17)
Others	0.82*(0.69,0.97)	0.82*(0.69,0.97)	0.82*(0.69,0.97)	0.82*(0.69,0.97)
**Working status**				
Working	Ref.	Ref.	Ref.	Ref.
Retired	1.58*(1.47,1.69)	1.58*(1.47,1.69)	1.58*(1.47,1.69)	1.58*(1.47,1.69)
Not working	1.59*(1.46,1.74)	1.59*(1.46,1.73)	1.59*(1.46,1.74)	1.59*(1.46,1.74)
**Tobacco consumption**				
No	Ref.	Ref.	Ref.	Ref.
Yes	0.95(0.9,1.01)	0.95(0.9,1.01)	0.95(0.9,1.02)	0.95(0.9,1.01)
**Alcohol consumption**				
No	Ref.	Ref.	Ref.	Ref.
Yes	0.97(0.9,1.06)	0.97(0.9,1.06)	0.97(0.9,1.06)	0.97(0.9,1.06)
**MPCE**				
Poorest	Ref.	Ref.	Ref.	Ref.
Poorer	1.19*(1.10,1.30)	1.19*(1.11,1.30)	1.19*(1.10,1.33)	1.19*(1.1,1.3)
Middle	1.30*(1.19,1.41)	1.31*(1.19,1.41)	1.32*(1.20,1.41)	1.3*(1.19,1.41)
Richer	1.47*(1.35,1.6)	1.47*(1.35,1.6)	1.47*(1.35,1.6)	1.47*(1.35,1.6)
Richest	1.64*(1.50,1.79)	1.64*(1.5,1.79)	1.64*(1.5,1.79)	1.64*(1.5,1.79)
**Religion**				
Hindu	Ref.	Ref.	Ref.	Ref.
Muslim	1.31*(1.21,1.43)	1.31*(1.21,1.43)	1.31*(1.21,1.43)	1.31*(1.21,1.43)
Christian	1.20*(1.08,1.34)	1.20*(1.08,1.34)	1.20*(1.08,1.34)	1.2*(1.08,1.34)
Others	1.30*(1.15,1.47)	1.30*(1.15,1.47)	1.30*(1.15,1.47)	1.3*(1.15,1.47)
**Caste**				
Scheduled Caste	Ref.	Ref.	Ref.	Ref.
Scheduled Tribe	0.66*(0.59,0.74)	0.66*(0.59,0.74)	0.66*(0.59,0.74)	0.66*(0.59,0.74)
Other Backward Class	1.02(0.94,1.10)	1.02(0.94,1.10)	1.03(0.94,1.10)	1.02(0.94,1.10)
Others	1.08(0.99,1.18)	1.08(0.99,1.18)	1.08(0.99,1.18)	1.08(0.99,1.18)
**Place of residence**				
Rural	Ref.	Ref.	Ref.	Ref.
Urban	1.34*(1.26,1.42)	1.34*(1.26,1.42)	1.34*(1.26,1.42)	1.34*(1.26,1.42)
**Region**				
North	Ref.	Ref.	Ref.	Ref.
Central	0.61*(0.55,0.68)	0.61*(0.55,0.68)	0.61*(0.56,0.68)	0.61*(0.55,0.68)
East	0.98(0.89,1.07)	0.98(0.89,1.07)	0.98(0.89,1.07)	0.98(0.90,1.07)
Northeast	1.00(0.89,1.12)	1.00(0.89,1.12)	1.00(0.89,1.12)	1.00(0.89,1.12)
West	1.07(0.97,1.17)	1.07(0.97,1.17)	1.07(0.97,1.18)	1.07(0.97,1.17)
South	1.22*(1.12,1.33)	1.22*(1.12,1.33)	1.22*(1.12,1.33)	1.22*(1.12,1.33)
**Obese/overweight** # **Physical activity status**				
Yes # frequent		Ref.		
No # frequent		0.58*(0.50,0.68)		
No # rare		0.60*(0.51,0.70)		
No # never		0.72*(0.63,0.83)		
Yes # rare		0.93(0.76,1.13)		
Yes # never		1.14(0.99,1.32)		
**High risk waist circumference** # **Physical activity status**				
Yes # frequent			Ref.	
No # frequent			0.69*(0.58,0.81)	
No # rare			0.67*(0.57,0.8)	
No # never			0.83*(0.71,0.97)	
Yes # rare			1.07(0.87,1.32)	
Yes # never			1.24*(1.07,1.45)	
**High risk waist-Hip ratio** # **Physical activity status**				
Yes # frequent				Ref.
No # frequent				0.75*(0.63,0.89)
No # rare				0.64*(0.52,0.78)
No # never				0.93(0.84,1.04)
Yes # rare				1.04(0.93,1.16)
Yes # never				1.21*(1.12,1.32)

Ref: Reference category;

^#^: Interaction;

*if p<0.05;

AOR: Adjusted odds ratio.

The likelihood of CVD was 60 per cent, 50 per cent, and 34 per cent significantly more likely among older adults who were obese/overweight [AOR: 1.60; CI: 1.48–1.72], had high risk waist circumference [AOR: 1.50; CI: 1.39–1.62] and suffered with high-risk waist-Hip ratio [AOR: 1.34; CI: 1.25–1.44], respectively compared to older adults with normal BMI and those who do not have a high risk waist circumference and high risk waist hip ratio. Moreover, older adults who never did physical activity were 22 per cent more likely to suffer from CVD than those who did frequent [AOR: 1.22; CI: 1.13–1.32]. The likelihood of CVD was 22 per cent and 19 per cent higher among older adults who belonged to 70–79 years [AOR: 1.22; CI: 1.15–1.30] and 80+ years age group [AOR: 1.19; CI: 1.08–1.30] respectively compared to those who belonged 60–69 years. Similarly, the likelihood of CVD was significantly higher in urban areas than rural counterparts [AOR: 1.34; CI: 1.26–1.42].

Interaction analysis shows that older adults who were not obese/overweight and did frequent physical activity had lower odds of CVD compared to obese/overweight older adults who did frequent physical activity [AOR: 0.58; CI: 0.50–0.68]. Similarly, older adults who were not obese/overweight [AOR: 0.60; CI: 0.51–0.70] and either rarely did or never did any physical activity [AOR: 0.72; CI: 0.63–0.83] had lower likelihood of CVD. With respect to older adults who had high risk waist circumference and did frequent physical activity, older adults who had high risk waist circumference and never did physical activity were 24 per cent more likely to suffer from CVD [AOR: 1.24; CI: 1.07–1.45]. Moreover, older adults who had high risk waist-Hip ratio and never did physical activity were 21 per cent more likely to suffer from CVD compared to those who had high risk waist-Hip ratio and did frequent physical activity [AOR: 1.21; CI: 1.12–1.32].

## Discussion

The demographic transition accompanied by the epidemiological transition in India has not only led to an increase in the geriatric population but also a shift from communicable diseases to non-communicable diseases [38]. Cardiovascular disease (CVD) is the leading cause of death in most countries worldwide. Obesity is a chronic metabolic disorder associated with cardiovascular disease risk, increased morbidity and early mortality. Currently, obesity has been considered to have reached a pandemic level globally and is found across all age groups [22, 39]. In view of the current situation, our study hypothesized an association between anthropometric measures of obesity, i.e. BMI and WHR and risk of CVD among older adults. We further tried to find the association between physical activity and CVD among older adults aged 60 and above. The study finds that BMI level is an important predictor of CVD among older adults in India. The measures of obesity, i.e. high risk waist circumference (WC) and high risk waist- hip ratio (WHR) are the major determinants of CVD among the geriatric population. Physical activity status is also an important determinant of CVD. Urban place of residents, work status and MPCE quintile are the major socio-economic factors which affect the risk of CVD among older adults in India.

Over the past three decades, the prevalence of the CVD risk factors hypertension and raised plasma cholesterol levels have declined in many high-income countries, but the prevalence of overweight and obesity continued to increase in both developed and developing countries [40]. Overweight/obesity in turn acted as a major determinant of health risks [40]. Our finding that obesity is a risk factor of CVD among the older adult population has several theoretical validations. Earlier studies have indicated that obesity is associated with reduced physical activity and several chronic conditions including type 2 diabetes, hypertension, and dyslipidaemia which lead to a high prevalence of CVD [41]. A study on older adult population in Ghana showed a similar finding that Ghanaians aged 60 and above who were overweight and obese had a higher risk of stage 1 and stage 2 hypertension, and were more likely to be diagnosed with arthritis and report severe deficiencies with instrumental activities of daily living, i.e. higher risk of CVD [42]. Ageing in man is associated with considerable changes in body composition, like an increase in fat mass and lower height due to compressed vertebral bodies, kyphosis and osteoporosis, which are the causes of increased morbidity and mortality [43, 44]. Though increased BMI results in an increasing risk of CVD [45], but BMI is not a very accurate measure of obesity and does not always reflect as a major cause of disease burden [46, 47].

The other anthropometric measures of obesity i.e. waist circumference and waist hip ratio are more accurate measure of abdominal obesity which is a leading cause of CVD risk factors [48]. The study finding that with high risk waist circumference and high risk waist to hip ratio (WHR) the risk of CVD increases are in line with earlier finding based on the Chinese elderly population which showed that WHR was strongly associated with CVD and its risk factors [45]. A study on the effect of obesity on vascular stiffness among older adults indicated that excess body weight has both short- and long-term effects on the vascular system, and this might be one mechanism by which obesity is associated with cardiovascular disease [49]. There lies a disagreement on the topic of whether BMI or the distribution of adipose tissue as measured by WC and WHR confers the greater risk of developing CVD. Some studies have indicated that generalized obesity, usually assessed by the body mass index (BMI) expressed in kg/m2, adequately predicts the development of CVD or CVD associated death [50, 51]. In contrast, other studies believe that central or abdominal obesity is the key abnormality leading to cardiovascular pathology [52, 53]. But, in general, both BMI and other measures of obesity have an association with increased risk of CVD [54].

Studies in the Indian context have also shown results which are similar to our findings. The prevalence of CVD have increased in India compared to other developing countries and this phenomena is observed across all age group from young adults to older adults [9]. Previous literature on association of central obesity measure and metabolic risk factors for coronary heart disease (CHD) concluded that obesity measures like waist hip ratio and waist circumference are important predictors of CHD [55]. A study based on the effect of obesity on cardiovascular risk factors in urban population of South India found that overweight and obesity were the major risk factors for higher values of blood pressure, cholesterol, triglyceride and low-density lipoprotein cholesterol (LDL-C) [56].

Our finding that physical activity had an association with CVD risk is also consistent with earlier findings from prospective study that indicate an inverse relation between physical activity and risk of CVD [57, 58]. A study based on Finnish men and women also indicated similar finding that regular physical activity decreased the risk of CVD and related mortality among Finnish men and women [59]. Studies in the Indian context have also shown that sedentary lifestyles were associated with an increase in risk of CVD and 35–40 minutes per day of brisk walking was associated with over a 50% reduction in risk for coronary heart disease [60].

Other risk factors associated with CVD included the economic status and residence of the elderly respondents. Recent studies have indicated, changing lifestyle, urbanisation, sedentary lifestyle as factors associated with an increase in the CVD and this is found more among people belonging to the rich strata [4, 61, 62]. A study on CVD risk factors among urban and rural population aged 20–70 years indicated the disease risk to be more among the urban population. The factors which caused the rural urban difference included greater income and literacy, dietary fat, low physical activity and obesity [63].

The present study included data from recently available Longitudinal Ageing Study in India (LASI) which caters to older adult across the country. Thus, it is the first attempt to explore the association between obesity measures and CVD risk among older adults in India. However, it suffers from a major limitation that the data is cross-sectional data which cannot prove that abdominal obesity as measured by waist circumference and waist hip ratio is a causative risk factor for CVD. Moreover, the CVD risk was based on verbal recall of the respondents/patients which may indicate biased results.

## Conclusion

The burden of overweight and obesity, physical inactivity among the older adults increases the risk of CVD. Since overweight and obesity are significantly associated with increased risk of CVD, overweight and obesity among not only older adults but the entire population is a serious issue for consideration. Our findings highlight the urgent need for framing direct and indirect strategies to control the obesity epidemic in future to minimise the burden of CVD among older adults in India. Moreover, stress should be made on increasing the physical activities among older population. However, the presence of such a strong association could have major biological, clinical, and/or epidemiological implications. Further studies are indicated to explore the underlying mechanisms, causality, and possible epidemiological consequences of obesity and CVD.
